# Supplementary figures and images for: Towards modeling phage therapy
Source: PLoS Comput Biol. 2026 Jun 22;22(6):e1014408. doi: 10.1371/journal.pcbi.1014408 (PMC13298995; doi:10.1371/journal.pcbi.1014408)

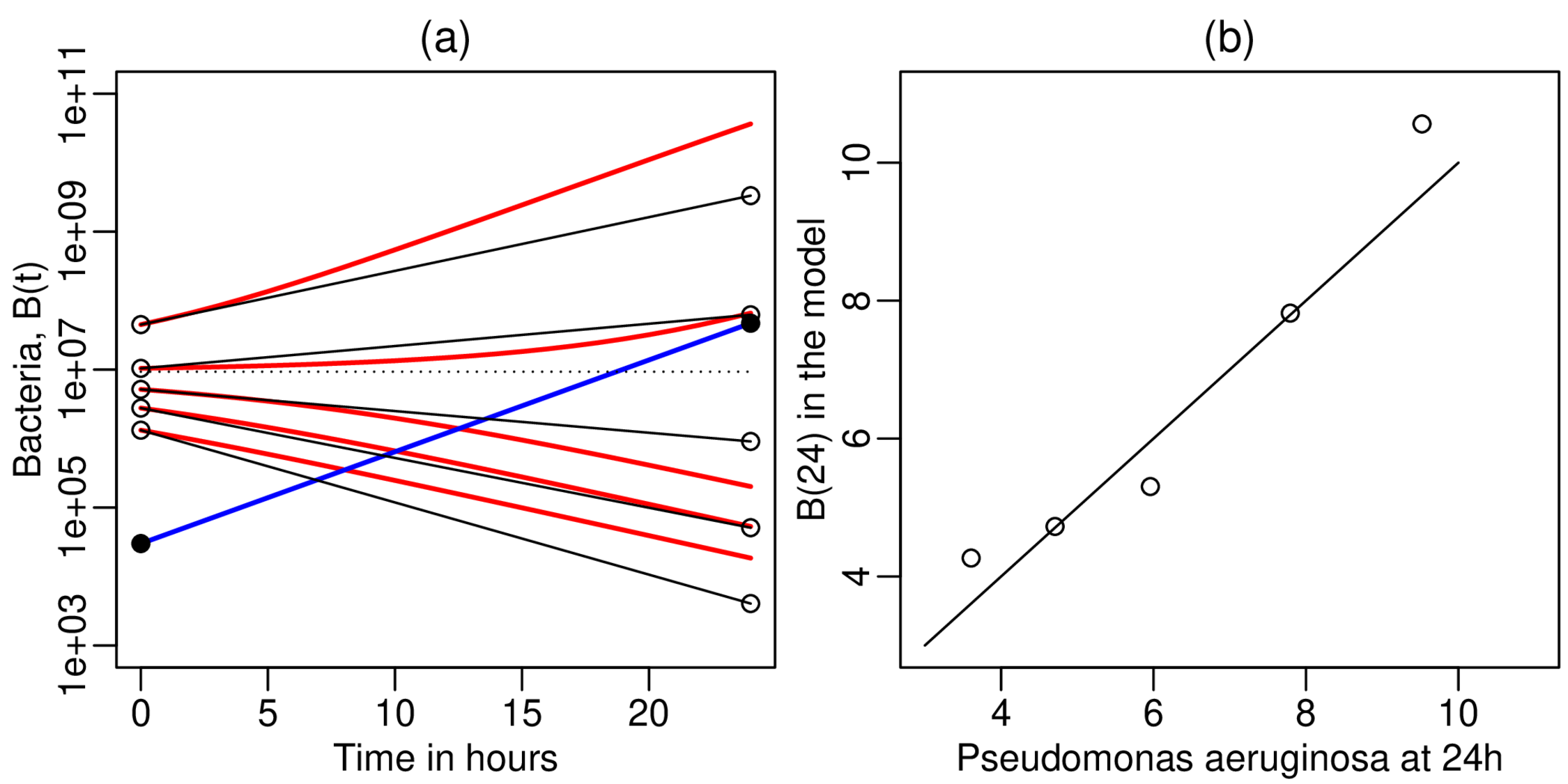

Supplement: S1 Fig — In Panel A the blue line depicts an example of bacterial growth in neutropenic mice (the bullets depict the observed concentrations at t = 0 and t = 24h). Assuming exponential growth this suggests a growth rate of r = 0.306 h–1 (see the main text). The black solid lines in Panel A depict bacterial growth curves starting at 5 different initial densities (the black circles depict the observations). Using these initial densities, the final densities were fitted with the model of Eq. (15) using κ and hk as free parameters, i.e., for K=8.48×10^11^ CFU/g and r = 0.306 h–1, we estimate κ=0.5 and hk=1.5×10^7^ CFU/g. These predictions are shown as red lines in Panel A and as symbols in Panel B. The horizontal dotted line in Panel A depicts the CBC of Eq. (15), i.e., hk(κ/r−1), for these estimated parameters (which indeed separates densities that expand from those that contract). (TIFF) [file pcbi.1014408.s001.tiff]

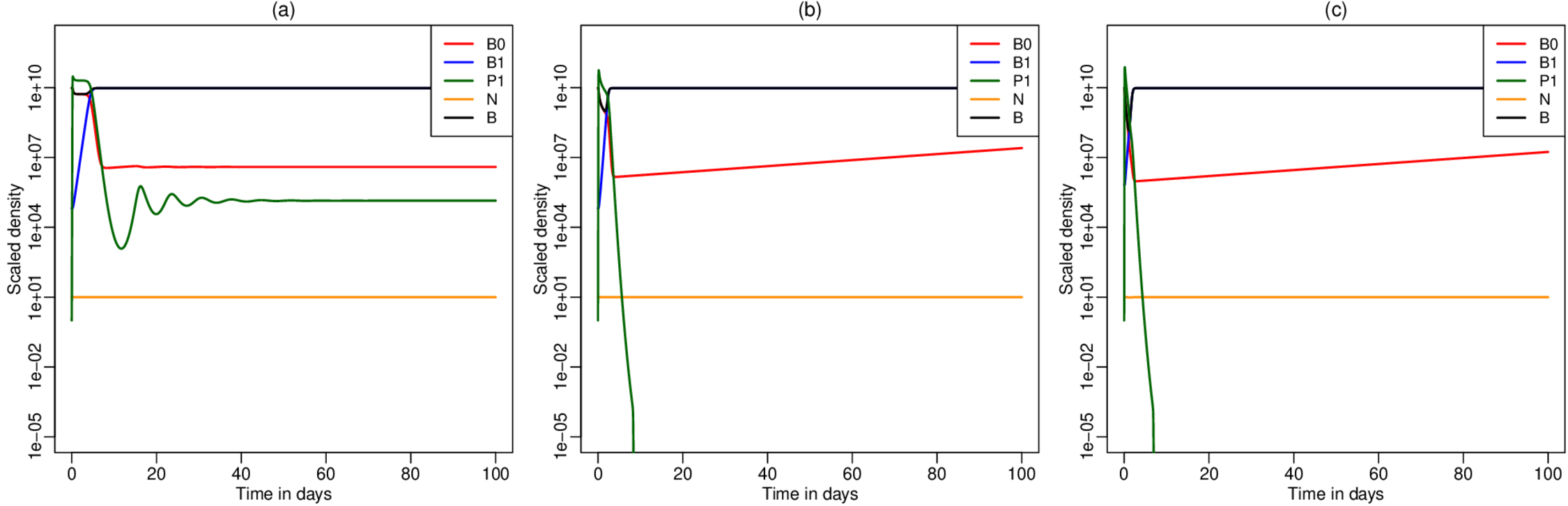

Supplement: S2 Fig — Panel A reveals that the phages recover following their initial decline, and that the system approaches a steady state with sensitive bacteria (B0), resistant bacteria (B1), and phages (P1) after a dampened oscillation that largely involves the phages and the sensitive bacteria. Panels B and C reveal that the phases go extinct during their initial decline (i.e., their densities drop below the threshold θ corresponding to about one phage per 10 kg of tissue). (TIFF) [file pcbi.1014408.s002.tiff]

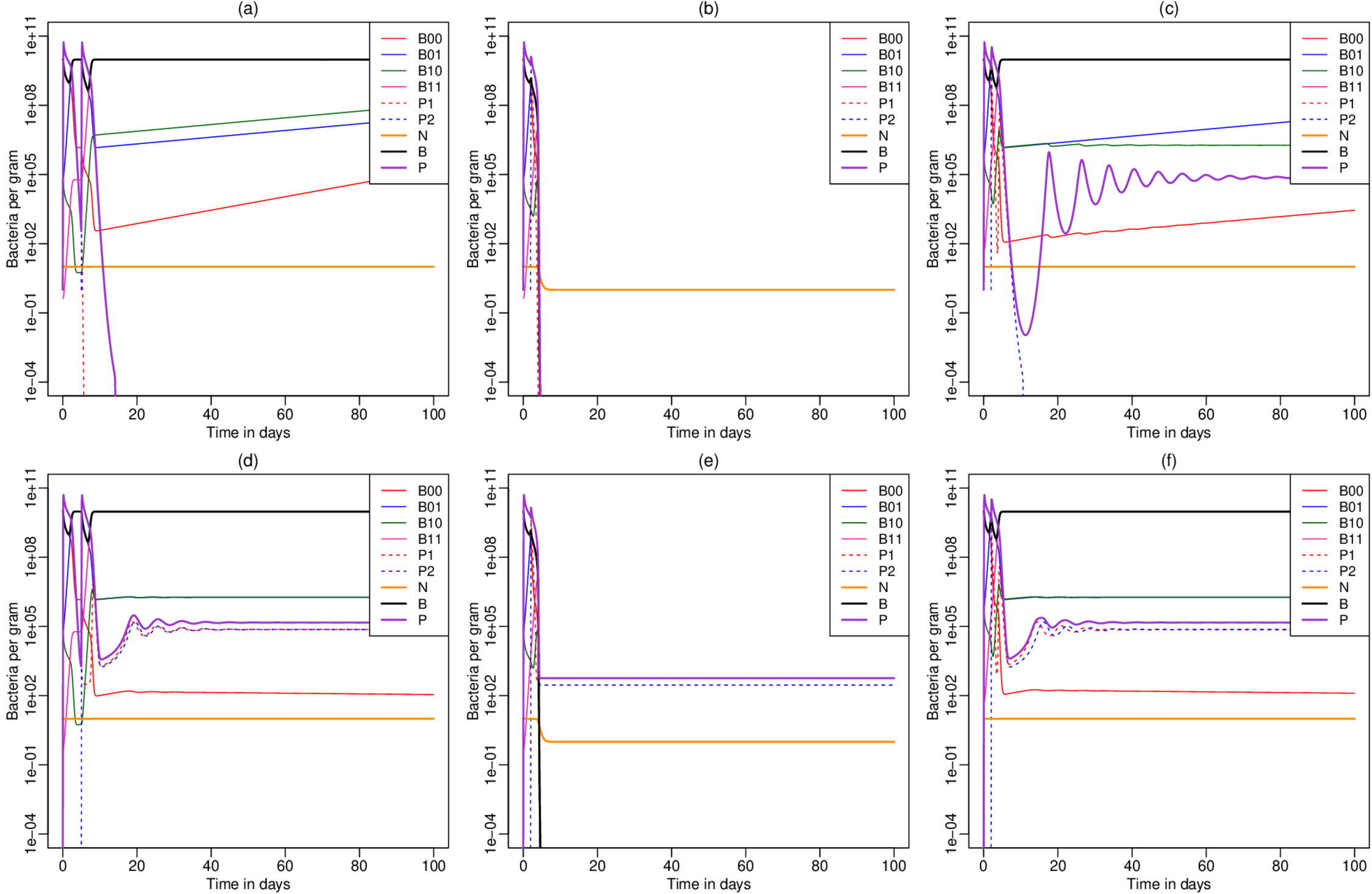

Supplement: S3 Fig — The phages go extinct in Panels A and B and persist in Panels D and F. Phages go extinct in Panel A because their density drops below the extinction threshold, θ, when the fully resistant bacteria take over. They go extinct in Panel B because the bacteria are cleared. In Panel E where the bacterial infection is also cleared, the phages persist at a steady state determined by the ratio of infusion and loss (i.e., P¯i=V¯/dP). In Panels C, D and F, where the resistant bacteria breach the upper CBC, the phages largely persist by infecting a minor population of sensitive bacteria (this steady state is again approached via a dampened oscillation). (TIFF) [file pcbi.1014408.s003.tiff]
