# Supplementary material for: Towards modeling phage therapy
Source: PLoS Comput Biol. 2026 Jun 22;22(6):e1014408. doi: 10.1371/journal.pcbi.1014408 (PMC13298995; doi:10.1371/journal.pcbi.1014408)
Supplement: Attachment — Submitted filename: new_cover_letter.pdf [file pcbi.1014408.s005.pdf]

Dear editors, dear Roland,

Thank you for inviting us to submit a revised version of our manuscript, and thanks for finding 3 expert reviewers. We have revised the manuscript and some of the figures along the excellent suggestions by the referees. We have added a discussion of the papers they have suggested, and we think this has markedly improved the quality of the manuscript.

Below please find our response (in red) to the questions and recommendations in a point-by-point manner. We how this revision suffices for making a favorable decision.

Kind regards

Rob de Boer, Robert Schooley & Alan Perelson

Reviewer #1: The authors build upon an existing mouse model of phage therapy that incorporates immune system synergy, extending it to a human context. The new model includes the use of phage cocktails and accounts for the potential of bacteria to independently evolve resistance to all cocktail components. The modeling approach and parameterization are thoughtfully designed and presented with meticulous clarity, making the work easy to follow and reproduce. Well-parameterized human models are critical for advancing phage therapy development, and this manuscript represents a valuable and timely contribution. I have mostly minor comments, listed below.

Thank you for this very positive feedback

Model:
Would cocktails still always be advantageous if phages might interfere with each other during infection?

No, cocktails will be less efficient if phages interfere. We have added that it would be best to include functionally different phages in the cocktails in lines 481-483 and 641 (and refer to Wright et al 2021). In the manuscript we also describe that bacteria can develop resistance to various cocktails at the same time (by shedding the envelope); see also lines 665-667.

L146: the latent period varies widely between phages and environments, how important is this assumption for the model results?

Using a latent period of half an hour, L=0.5 h, and a burst size of b=100 we estimate that there would be $b^2=10^4$ phages after an hour, and by solving $10^4=e^{rho}$ we obtain that rho=9.21 h^{-1}. Hence the formula is rho=ln[$b^{1/L}$]. If the latent period were L=1h we would obtain rho=4.6 h^{-1}, and if the burst size were b=1000, we would obtain rho=6.8 h^{-1}. So, the replication rate is typically fast. We indeed assume that phages replicate rapidly (and hence that the initial dose is not important), and that seems a safe assumption. We have added this to footnote 1.

L 330: Wouldn't the argument here be similar to the one later in the manuscript where more infectious phages lead to easier resistance evolution? A higher number of phages from the beginning would lead to a similar effect, meaning that the phage number does matter (arguing for a lower initial phage dose).

That is an interesting thought, but because the phages typically replicate so fast (they approach peak values on a time scale of a few hours; see Fig 2f) the initial dose hardly matters. Instead, if phages are more infectious, they reduce bacterial densities more strongly, leading to bacterial growth, and hence faster mutations.

L 345: While probably outside of this manuscript, it would be nice to also consider would partial cross-resistance in a future version of the model.

True, the model now has a binary matrix, M, to define resistance. It would be a good follow-up to extend it with partial resistance, and we now mention this in the discussion (line 689).

Antibiotics were included as a potential death rate for bacteria but they could also have an influence on phage numbers and infectivity through bacterial physiology and killing of phage-infected hosts.

Good point, we have added that antibiotics can also affect infected bacteria in Eq. 18. Since we haven't used the antibiotic treatment that does not affect the results, but this does make the model more complete.

L421: I am confused as to how the adsorption rate a is related to the infectivity vectors beta_j

We indeed write that this is a simplification, as we assume that phages also bind bacteria that they cannot infect. Binding the receptor is not sufficient for infection. But, indeed, if bacterial resistance is due to downregulation of the receptor, this general absorption term is too simple (which we admit). We added this explanation (line 390-392). Thanks.

Figures:
Fig.1 It might be a bit confusing t only see the sensitive bacteria appear in panel C. Maybe a red S could be written next to the total bacterial line as well to make clear that they are just not visible. And this could be mentioned in the legend. d) The difference between the three lines is hard to see, especially for the neutrophils, maybe changing the line types could help.

True, we added an S

Fig.2 it feels like switching a and b would allow a more logical ordering

Thanks, we have switched the panels.

Fig.4 B11 seems to have the same colour as N?

True, we have changed the color of B11.

Fig.5 It would be helpful to show the administration of cocktails 1 and 2 more clearly in the figure.

Thanks, we have added color-matched bold vectors and lines to indicate the administration of cocktails.

Discussion: The results could be linked back more to previous findings on phage cocktail efficacy, e.g. https://doi.org/10.1099/mic.0.001110, https://doi.org/10.1128/AEM.00757-12 and phage dosing, e.g. https://doi.org/10.1093/cid/ciad516

Thanks, these are good references that we now discuss and cite in several places.

Writing:
While it is very helpful to have a very detailed description of each model component and parametrization step, I am wondering if it would be helpful to separate them out a bit more into Methods and Results parts to improve the flow. E.g. 2.1 seems less of a result than a detailed methods section and could probably be given as a much shorter version in the Results section and an extended one in the Methods.

Thanks, we have moved the section on the initial replication rate, and on the neutrophil killing rate to the SI.

L 228: dB/dT = 0?

Typo is corrected.

L482: something seems weird about this sentence

True, the decline was a typo.

Reviewer #2: This is a very clear contribution that makes a compelling case for several key take-home messages. The authors have carefully justified their modelling assumptions throughout, and gone to great lengths to find and explore realistic parameter values. I particularly enjoyed the straight-forward "what have we learned" summaries on the way through the paper. The contribution is a model for writing up results with a clear eye to what we can (and cannot) reliably infer from mathematical models.

Thank you for this very positive feedback

Minor points:

1. It would be helpful to the reader to mention that the dN/dt equation will be coming later, when the S-R-P system is introduced (perhaps I just missed this). So around eqns 1-3 since (1) depends on N, or around eqns 10-13.

As suggested, we have moved the original dN/dt equation up (Now Eq 4).

2. I recognize that the SI is already substantial. Nonetheless I found the paper somewhat overly detailed in the parameter estimate section. Table 1 and the associated text, for example, are of interest to readers who are *very* close to this work, whereas the figures for example will be of quite broad interest. The Discussion and Introduction are nicely aimed for broad appeal. Perhaps a little more of the parameter estimation could be directed to the SI, with a broader readership in mind for the main paper?

Thanks, we have moved the section on the initial replication rate, and on the neutrophil killing rate to the SI.

3. Clarify that the A_j are constants?

Good point, we added that (line 387).

4. Figure 3 should be re-arranged such that panels (a) and (b) are in a column, followed by (c & d), then (e & f). Or re-arrange as (3x2) rather than (2x3).

That is indeed more clear: done.

Typos, etc:

1. line 219 "These data"

Fixed.

2. atto-fox: I'm very familiar with this phenomenon, but is it sufficiently well-known for your intended readership? Maybe there's an atto-fox reference you could give for the new reader (eg. grad student who has no idea what this might mean)?

Good point, we added a reference (line 405).

3. line 682, double negative "Not all ... need not"

Fixed.

Reviewer #3: Review of Boer et al. "Modeling phage therapy"

The manuscript by Boer and colleagues is a highly thorough consideration of the bridge between theoretical population dynamic simulations and real-world mouse and patient data. First, the authors develop an ODE model, based on a previously published model by Roach and colleagues, which accounts for the role of the host response (neutrophils) in the dynamics of a single phage treatment of one bacterial population which can evolve resistance. This adapted model is parametrized and ground-truthed against the same mouse data as the Roach model. Next, the authors attempt to retrospectively explain patient outcomes during phage therapy by further updating their model to account for multiple phages and numerous bacterial states. Finally, the authors interpret their simulations to make suggestions about how to successfully employ phage for therapy. Overall, this manuscript represents a strong and important contribution to the field of phage therapy and more generally to the quantitative modeling of antimicrobial treatment and is a good fit for publication in PLOS Computation Biology. That being said, I do have suggestions that the authors should consider in improving their manuscript prior to acceptance.

Thank you for this very positive feedback

Major Comments:

1. While the authors use the common variables in the field of phage modeling, they are not defined until Table S2. In contrast the states are defined in the text in the first paragraph of the results. This manuscript has the potential to be of interest to mathematical biologists that do

not work with phage; thus, the authors should define the variables prior to using them, most likely with a table similar to S2.

Good point, we have added a sentence in the main text saying that parameter names and definitions are described in Table S.2 (now Table S.3 in line 394. Since other referees recommend moving more technical details to the SI, we prefer to leave Table S2 in the SI.

2. The authors intend to employ this model generally to phage therapy, thus they should perform a sensitivity analysis for at least some critical parameters which are likely to vary such as b, μ (outside of the context of the multiphage model), k, and the CBC threshold. This analysis also opens up further interpretation by the authors as to what makes a good phage (e.g., if we see the model is very sensitive to a phage specific parameter, then we should consider selecting phage to optimize for that parameter).

We do extensively discuss that we see our model as a new framework enabling one to study phage therapy, and that we see our simulation as examples of mechanisms that are most likely to have played a role. We think that similar examples can be found for various other parameter values. In our answer to the first reviewer, we point out that the burst size and the replication rate will typically lead to fast replication of phages (added to Footnote 1). In Figure 6 we perform a parameter sweep on the infection rate, and we conclude that the infection rate hardly matters once it exceeds the critical infection rate. We now emphasize that result more by referring to the Wright et al paper, and thereby re-emphasize that selection for cocktails with functionally different phages is more important than optimizing the individual phages (line 625-627).

3. A more direct and thorough consideration of the limitations of the application of their model (critically, the things their model does not consider) should be brought up in the discussion. This section should be suited for those in the field who are not as familiar with mathematical and computer-simulation modelling.

In the discussion, we already wrote that our model remains a caricature of the complex reality. We have added that our model lacks other immune responses like macrophages, complement system, antibodies and T cells (lines 680-682). We added that allowing for partial resistance would be good (lines 689-690).

4. The authors should also expand the second to last paragraph of the discussion to more thoroughly consider what sort of empirical data would further the field based on the predictions of their modelling. The authors should consider both experimental in vitro and clinical data that would be useful. It would not be inappropriate to suggest specific experiments to either further support or reject the predictions of their model.

Thanks, we have expanded that paragraph (lines 689-699).

5. While the authors plausibly explain the biology (phage defense systems) of why the pretreatment resistance frequency is so high in the Patterson case, how can they explain the biology of the incredibly high mutation rate which they use in Figures 5 and 6 (versus the more reasonable rate used in Figure 3)?

We already write in line 663 that the high pretreatment frequencies of resistant bacteria could be the result of a long evolutionary arms race. We added that the shedding of the envelope is could be an example of such a bacterial adaptation (line 665-667)

Minor Comments:

1. While the manuscript certainly does model phage therapy, the title feels a bit ambitious as if it were the definitive manuscript on modeling phage therapy. A slightly more descriptive title should be considered.

How about: Towards modeling phage therapy?

2. On my printed copy of the manuscript, the line thickness varies substantially between figures which makes the later figures (e.g., Figure 5 and Figure S3) difficult to interpret.

Thanks, the line thickness in some of the legends was indeed not consistent with those of the graphs. We now give every line a unique color and correctly indicate the line thickness in the legend. Only the totals are heavy.

3. I'd suggest another term instead of "Guessed" for the assumed parameter values in Table S2.

We have changed this into Estimated.

4. The ReadMe for the Zenodo data is not particularly helpful but for recreating the figures.

We have added some info to the ReadMe, but also think that having fairly straightforward code that recreates the figures should enable anyone to repeat the numerical analyses.
