# Supplementary material for: Towards modeling phage therapy
Source: PLoS Comput Biol. 2026 Jun 22;22(6):e1014408. doi: 10.1371/journal.pcbi.1014408 (PMC13298995; doi:10.1371/journal.pcbi.1014408)
Supplement: Peer review history

# Peer review: Decision Letter 0

**DOI:** 10.1371/journal.pcbi.1014408.r001

**Authors:** Roland Regoes, James Faeder

5 Feb 2026

PCOMPBIOL-D-25-02336

Modeling phage therapy

PLOS Computational Biology

Dear Dr. De Boer,

Thank you for submitting your manuscript to PLOS Computational Biology. After careful consideration, we feel that it has merit but does not fully meet PLOS Computational Biology's publication criteria as it currently stands. Therefore, we invite you to submit a revised version of the manuscript that addresses the points raised during the review process.

Please submit your revised manuscript by Apr 07 2026 11:59PM. If you will need more time than this to complete your revisions, please reply to this message or contact the journal office at ploscompbiol@plos.org. When you're ready to submit your revision, log on to https://www.editorialmanager.com/pcompbiol/ and select the 'Submissions Needing Revision' folder to locate your manuscript file.

Please include the following items when submitting your revised manuscript:

* A letter that responds to each point raised by the editor and reviewer(s). You should upload this letter as a separate file labeled 'Response to Reviewers'. This file does not need to include responses to formatting updates and technical items listed in the 'Journal Requirements' section below.

* A marked-up copy of your manuscript that highlights changes made to the original version. You should upload this as a separate file labeled 'Revised Manuscript with Track Changes'.

* An unmarked version of your revised paper without tracked changes. You should upload this as a separate file labeled 'Manuscript'.

If you would like to make changes to your financial disclosure, competing interests statement, or data availability statement, please make these updates within the submission form at the time of resubmission. Guidelines for resubmitting your figure files are available below the reviewer comments at the end of this letter.

We look forward to receiving your revised manuscript.

Kind regards,

Roland R Regoes

Academic Editor

PLOS Computational Biology

James Faeder

Section Editor

PLOS Computational Biology

**Journal Requirements:**

1) We ask that a manuscript source file is provided at Revision. Please upload your manuscript file as a .doc, .docx, .rtf or .tex. If you are providing a .tex file, please upload it under the item type u2018LaTeX Source Fileu2019 and leave your .pdf version as the item type u2018Manuscriptu2019.

2) Please provide an Author Summary. This should appear in your manuscript between the Abstract (if applicable) and the Introduction, and should be 150-200 words long. The aim should be to make your findings accessible to a wide audience that includes both scientists and non-scientists. Sample summaries can be found on our website under Submission Guidelines:

https://journals.plos.org/ploscompbiol/s/submission-guidelines#loc-parts-of-a-submission

3) Please upload all main figures as separate Figure files in .tif or .eps format. For more information about how to convert and format your figure files please see our guidelines:

https://journals.plos.org/ploscompbiol/s/figures

4) We notice that your supplementary Figures, Tables, and information are included in the manuscript file. Please remove them and upload them with the file type 'Supporting Information'. Please ensure that each Supporting Information file has a legend listed in the manuscript after the references list.

5) Please amend your detailed Financial Disclosure statement. This is published with the article. It must therefore be completed in full sentences and contain the exact wording you wish to be published.

- State the initials, alongside each funding source, of each author to receive each grant. For example: "This work was supported by the National Institutes of Health (####### to AM; ###### to CJ) and the National Science Foundation (###### to AM)."

- State what role the funders took in the study. If the funders had no role in your study, please state: "The funders had no role in study design, data collection and analysis, decision to publish, or preparation of the manuscript.".

If you did not receive any funding for this study, please simply state: u201cThe authors received no specific funding for this work.u201d

**Reviewers' comments:**

Reviewer's Responses to Questions

**Comments to the Authors:**

**Please note here if the review is uploaded as an attachment.**

Reviewer #1: The authors build upon an existing mouse model of phage therapy that incorporates immune system synergy, extending it to a human context. The new model includes the use of phage cocktails and accounts for the potential of bacteria to independently evolve resistance to all cocktail components. The modeling approach and parameterization are thoughtfully designed and presented with meticulous clarity, making the work easy to follow and reproduce. Well-parameterized human models are critical for advancing phage therapy development, and this manuscript represents a valuable and timely contribution. I have mostly minor comments, listed below.

Model:

Would cocktails still always be advantageous if phages might interfere with each other during infection?

L146: the latent period varies widely between phages and environments, how important is this assumption for the model results?

L 330: Wouldn’t the argument here be similar to the one later in the manuscript where more infectious phages lead to easier resistance evolution? A higher number of phages from the beginning would lead to a similar effect, meaning that the phage number does matter (arguing for a lower initial phage dose).

L 345: While probably outside of this manuscript, it would be nice to also consider would partial cross-resistance in a future version of the model.

Antibiotics were included as a potential death rate for bacteria but they could also have an influence on phage numbers and infectivity through bacterial physiology and killing of phage-infected hosts.

L421: I am confused as to how the adsorption rate a is related to the infectivity vectors beta_j

Figures:

Fig.1 It might be a bit confusing t only see the sensitive bacteria appear in panel C. Maybe a red S could be written next to the total bacterial line as well to make clear that they are just not visible. And this could be mentioned in the legend. d) The difference between the three lines is hard to see, especially for the neutrophils, maybe changing the line types could help.

Fig.2 it feels like switching a and b would allow a more logical ordering

Fig.4 B11 seems to have the same colour as N?

Fig.5 It would be helpful to show the administration of cocktails 1 and 2 more clearly in the figure.

Discussion: The results could be linked back more to previous findings on phage cocktail efficacy, e.g. https://doi.org/10.1099/mic.0.001110, https://doi.org/10.1128/AEM.00757-12 and phage dosing, e.g. https://doi.org/10.1093/cid/ciad516

Writing:

While it is very helpful to have a very detailed description of each model component and parametrization step, I am wondering if it would be helpful to separate them out a bit more into Methods and Results parts to improve the flow. E.g. 2.1 seems less of a result than a detailed methods section and could probably be given as a much shorter version in the Results section and an extended one in the Methods.

L 228: dB/dT = 0?

L482: something seems weird about this sentence

Reviewer #2: This is a very clear contribution that makes a compelling case for several key take-home messages. The authors have carefully justified their modelling assumptions throughout, and gone to great lengths to find and explore realistic parameter values. I particularly enjoyed the straight-forward "what have we learned" summaries on the way through the paper. The contribution is a model for writing up results with a clear eye to what we can (and cannot) reliably infer from mathematical models.

Minor points:

1. It would be helpful to the reader to mention that the dN/dt equation will be coming later, when the S-R-P system is introduced (perhaps I just missed this). So around eqns 1-3 since (1) depends on N, or around eqns 10-13.

2. I recognize that the SI is already substantial. Nonetheless I found the paper somewhat overly detailed in the parameter estimate section. Table 1 and the associated text, for example, are of interest to readers who are *very* close to this work, whereas the figures for example will be of quite broad interest. The Discussion and Introduction are nicely aimed for broad appeal. Perhaps a little more of the parameter estimation could be directed to the SI, with a broader readership in mind for the main paper?

3. Clarify that the A_j are constants?

4. Figure 3 should be re-arranged such that panels (a) and (b) are in a column, followed by (c & d), then (e & f). Or re-arrange as (3x2) rather than (2x3).

Typos, etc:

1. line 219 "These data"

2. atto-fox: I'm very familiar with this phenomenon, but is it sufficiently well-known for your intended readership? Maybe there's an atto-fox reference you could give for the new reader (eg. grad student who has no idea what this might mean)?

3. line 682, double negative "Not all ... need not"

Reviewer #3: Review of Boer et al. “Modeling phage therapy”

The manuscript by Boer and colleagues is a highly thorough consideration of the bridge between theoretical population dynamic simulations and real-world mouse and patient data. First, the authors develop an ODE model, based on a previously published model by Roach and colleagues, which accounts for the role of the host response (neutrophils) in the dynamics of a single phage treatment of one bacterial population which can evolve resistance. This adapted model is parametrized and ground-truthed against the same mouse data as the Roach model. Next, the authors attempt to retrospectively explain patient outcomes during phage therapy by further updating their model to account for multiple phages and numerous bacterial states. Finally, the authors interpret their simulations to make suggestions about how to successfully employ phage for therapy. Overall, this manuscript represents a strong and important contribution to the field of phage therapy and more generally to the quantitative modeling of antimicrobial treatment and is a good fit for publication in PLOS Computation Biology. That being said, I do have suggestions that the authors should consider in improving their manuscript prior to acceptance.

Major Comments:

1. While the authors use the common variables in the field of phage modeling, they are not defined until Table S2. In contrast the states are defined in the text in the first paragraph of the results. This manuscript has the potential to be of interest to mathematical biologists that do not work with phage; thus, the authors should define the variables prior to using them, most likely with a table similar to S2.

2. The authors intend to employ this model generally to phage therapy, thus they should perform a sensitivity analysis for at least some critical parameters which are likely to vary such as b, µ (outside of the context of the multiphage model), k, and the CBC threshold. This analysis also opens up further interpretation by the authors as to what makes a good phage (e.g., if we see the model is very sensitive to a phage specific parameter, then we should consider selecting phage to optimize for that parameter).

3. A more direct and thorough consideration of the limitations of the application of their model (critically, the things their model does not consider) should be brought up in the discussion. This section should be suited for those in the field who are not as familiar with mathematical and computer-simulation modelling.

4. The authors should also expand the second to last paragraph of the discussion to more thoroughly consider what sort of empirical data would further the field based on the predictions of their modelling. The authors should consider both experimental in vitro and clinical data that would be useful. It would not be inappropriate to suggest specific experiments to either further support or reject the predictions of their model.

5. While the authors plausibly explain the biology (phage defense systems) of why the pretreatment resistance frequency is so high in the Patterson case, how can they explain the biology of the incredibly high mutation rate which they use in Figures 5 and 6 (versus the more reasonable rate used in Figure 3)?

Minor Comments:

1. While the manuscript certainly does model phage therapy, the title feels a bit ambitious as if it were the definitive manuscript on modeling phage therapy. A slightly more descriptive title should be considered.

2. On my printed copy of the manuscript, the line thickness varies substantially between figures which makes the later figures (e.g., Figure 5 and Figure S3) difficult to interpret.

3. I’d suggest another term instead of “Guessed” for the assumed parameter values in Table S2.

4. The ReadMe for the Zenodo data is not particularly helpful but for recreating the figures.

**********

**Have the authors made all data and (if applicable) computational code underlying the findings in their manuscript fully available?**

The PLOS Data policy requires authors to make all data and code underlying the findings described in their manuscript fully available without restriction, with rare exception (please refer to the Data Availability Statement in the manuscript PDF file). The data and code should be provided as part of the manuscript or its supporting information, or deposited to a public repository. For example, in addition to summary statistics, the data points behind means, medians and variance measures should be available. If there are restrictions on publicly sharing data or code —e.g. participant privacy or use of data from a third party—those must be specified.

Reviewer #1: Yes

Reviewer #2: Yes

Reviewer #3: Yes

**********

PLOS authors have the option to publish the peer review history of their article (what does this mean?). If published, this will include your full peer review and any attached files.

If you choose “no”, your identity will remain anonymous but your review may still be made public.

**Do you want your identity to be public for this peer review?**  For information about this choice, including consent withdrawal, please see our Privacy Policy.

Reviewer #1: No

Reviewer #2: No

Reviewer #3: No

[NOTE: If reviewer comments were submitted as an attachment file, they will be attached to this email and accessible via the submission site. Please log into your account, locate the manuscript record, and check for the action link "View Attachments". If this link does not appear, there are no attachment files.]

**Figure resubmission:**

-->While revising your submission, we strongly recommend that you use PLOS’s NAAS tool (https://ngplosjournals.pagemajik.ai/artanalysis) to test your figure files. NAAS can convert your figure files to the TIFF file type and meet basic requirements (such as print size, resolution), or provide you with a report on issues that do not meet our requirements and that NAAS cannot fix.

After uploading your figures to PLOS’s NAAS tool - https://ngplosjournals.pagemajik.ai/artanalysis, NAAS will process the files provided and display the results in the "Uploaded Files" section of the page as the processing is complete. If the uploaded figures meet our requirements (or NAAS is able to fix the files to meet our requirements), the figure will be marked as "fixed" above. If NAAS is unable to fix the files, a red "failed" label will appear above. When NAAS has confirmed that the figure files meet our requirements, please download the file via the download option, and include these NAAS processed figure files when submitting your revised manuscript.

**Reproducibility:**

To enhance the reproducibility of your results, we recommend that authors of applicable studies deposit laboratory protocols in protocols.io, where a protocol can be assigned its own identifier (DOI) such that it can be cited independently in the future. Additionally, PLOS ONE offers an option to publish peer-reviewed clinical study protocols. Read more information on sharing protocols at https://plos.org/protocols?utm_medium=editorial-email&utm_source=authorletters&utm_campaign=protocols

# Author comment: Author response to Decision Letter 1

**DOI:** 10.1371/journal.pcbi.1014408.r002

20 May 2026

Attachment Submitted filename: new_cover_letter.pdf

# Editor report: Decision Letter 1

**DOI:** 10.1371/journal.pcbi.1014408.r003

**Authors:** Roland Regoes, James Faeder, Roland Regoes, James Faeder

4 Jun 2026

Dear Prof. De Boer,

We are pleased to inform you that your manuscript 'Towards modeling phage therapy' has been provisionally accepted for publication in PLOS Computational Biology.

Before your manuscript can be formally accepted you will need to complete some formatting changes, which you will receive in a follow up email. A member of our team will be in touch with a set of requests.

Please note that your manuscript will not be scheduled for publication until you have made the required changes, so a swift response is appreciated.

IMPORTANT: The editorial review process is now complete. PLOS will only permit corrections to spelling, formatting or significant scientific errors from this point onwards. Requests for major changes, or any which affect the scientific understanding of your work, will cause delays to the publication date of your manuscript.

Should you, your institution's press office or the journal office choose to press release your paper, you will automatically be opted out of early publication. We ask that you notify us now if you or your institution is planning to press release the article. All press must be co-ordinated with PLOS.

Thank you again for supporting Open Access publishing; we are looking forward to publishing your work in PLOS Computational Biology.

Best regards,

Roland R Regoes

Academic Editor

PLOS Computational Biology

James Faeder

Section Editor

PLOS Computational Biology

***********************************************************

# Editor report: Acceptance letter

**DOI:** 10.1371/journal.pcbi.1014408.r004

**Authors:** Roland Regoes, James Faeder, Roland Regoes, James Faeder

PCOMPBIOL-D-25-02336R1

Towards modeling phage therapy

Dear Dr De Boer,

I am pleased to inform you that your manuscript has been formally accepted for publication in PLOS Computational Biology. Your manuscript is now with our production department and you will be notified of the publication date in due course.

The corresponding author will soon be receiving a typeset proof for review, to ensure errors have not been introduced during production. Please review the PDF proof of your manuscript carefully, as this is the last chance to correct any errors. Please note that major changes, or those which affect the scientific understanding of the work, will likely cause delays to the publication date of your manuscript.

Soon after your final files are uploaded, unless you have opted out, the early version of your manuscript will be published online. The date of the early version will be your article's publication date. The final article will be published to the same URL, and all versions of the paper will be accessible to readers.

For Research, Software, and Methods articles, you will receive an invoice from PLOS for your publication fee after your manuscript has reached the completed accept phase. If you receive an email requesting payment before acceptance or for any other service, this may be a phishing scheme. Learn how to identify phishing emails and protect your accounts at https://explore.plos.org/phishing.

Thank you again for supporting PLOS Computational Biology and open-access publishing. We are looking forward to publishing your work!

With kind regards,

Zsofia Freund

PLOS Computational Biology | Carlyle House, Carlyle Road, Cambridge CB4 3DN | United Kingdom ploscompbiol@plos.org | Phone +44 (0) 1223-442824 | ploscompbiol.org | @PLOSCompBiol
